# Determination of Voriconazole Plasma Concentration by HPLC Technique and Evaluating Its Association with Clinical Outcome and Adverse Effects in Patients with Invasive Aspergillosis

**DOI:** 10.1155/2021/5497427

**Published:** 2021-04-12

**Authors:** Sahar Yousefian, Farzaneh Dastan, Majid Marjani, Payam Tabarsi, Saghar Barati, Nahid Shahsavari, Farzad Kobarfard

**Affiliations:** ^1^Department of Clinical Pharmacy, School of Pharmacy, Shahid Beheshti University of Medical Sciences, Tehran, Iran; ^2^Chronic Respiratory Diseases Research Center, National Research Institute of Tuberculosis and Lung Diseases (NRITLD), Shahid Beheshti University of Medical Sciences, Tehran, Iran; ^3^Clinical Tuberculosis and Epidemiology Research Center, National Research Institute of Tuberculosis and Lung Diseases (NIRTLD), Shahid Beheshti University of Medical Sciences, Tehran, Iran; ^4^Department of Medicinal Chemistry, School of Pharmacy, Shahid Beheshti University of Medical Sciences, Tehran, Iran

## Abstract

**Purpose:**

Invasive aspergillosis is a prevalent fungal disease, especially in Asian countries with a high mortality rate. Voriconazole (VRZ) is the first choice for invasive aspergillosis treatment. Plasma concentration of this drug is unpredictable and varies among individuals. This variability is influenced by many factors leading to clinical implication. Therapeutic drug monitoring (TDM) may have a crucial role in the patients' treatment process. The HPLC method provides sufficient specificity and sensitivity for plasma VRZ concentration determination for TDM purposes of this drug.

**Methods:**

Patients who initiated oral or intravenous VRZ for invasive aspergillosis were enrolled in this study. Demographic characteristics and clinical data, outcome, and adverse effects were documented. For each patient, the plasma sample was collected under steady-state condition and analyzed using a validated HPLC method.

**Results:**

A total of 22 measurements were performed. Fifty percent of patients were out of the therapeutic range. From them, 27.27% and 22.73% were in subtherapeutic and supratherapeutic ranges (<1 *μ*g/mL and >5.5 *μ*g/mL), respectively. There was a significant correlation between VRZ plasma concentration and treatment outcomes (*P*=0.022). Treatment failure was five times higher than treatment success in those in the subtherapeutic range. Adverse effects were observed more frequently in patients with supratherapeutic concentrations compared to those with non-supratherapeutic levels. Furthermore, the mortality rate in patients experiencing treatment failure was 2.17 times higher than those with treatment success.

**Conclusions:**

TDM of VRZ plays an important role in better evaluation of efficacy and toxicity during treatment. Therefore, determination of the drug level may be of clinical significance.

## 1. Introduction

Prevalence and severity of invasive fungal infections have increased during the last two decades. In hospital settings, the second prevalent fungal infection is aspergillosis, and approximately 90% of the cases are caused by *A. fumigatus* [1]. Among fungal infections, invasive aspergillosis leads to significant morbidity and mortality [2, 3]. Invasive aspergillosis is a prevalent fungal disease, especially in Asian countries with a high mortality rate. Global incidence of invasive aspergillosis is 300,000 cases annually (50% of them are in Asian countries), and the mortality rate is 30–80% [4]. Voriconazole (VRZ) is a second-generation antifungal agent which is a broad-spectrum derivative of fluconazole [5, 6]. This agent is available as oral and intravenous (IV) formulations [7]. VRZ is the first choice for invasive aspergillosis treatment [1, 8]. The oral bioavailability, protein binding, and tissue penetration of VRZ are 96%, 58%, and 2–4.6 L/kg, respectively [9]. VRZ is metabolized by hepatic CYP450 isoenzymes [2, 6] and has complicated and nonlinear pharmacokinetic characteristics [2]. The plasma concentration of this drug is unpredictable and varies within and between individuals [10] regardless of the route of administration [11]. This variability is influenced by many factors such as age, sex, body mass index (BMI), polymorphism of CYP2C19, drug-drug interaction, and underlying diseases [2, 9, 12], which leads to clinical implication [8]. As a result, therapeutic drug monitoring (TDM) is necessary to minimize toxicity and optimize efficacy [3, 5]. Indeed, VRZ is ideally appropriate for this approach [12], and TDM may have a crucial role in the patient's treatment process [6].

The plasma trough levels of VRZ are preferred for therapeutic monitoring [12]. Low VRZ trough levels (<1 *μ*g/ml) are correlated with therapeutic failure. High VRZ trough levels (>5.5 *μ*g/ml) are associated with toxicities including skin rash and photosensitivity reaction, visual disturbance, hallucination, hepatotoxicity, and central nervous system (CNS) events [7–9, 12]. Since TDM of VRZ plays an important role in better evaluation of efficacy and toxicity of treatment, determination of the drug level may be of clinical significance.

Many analytical techniques such as high-performance liquid chromatography (HPLC), liquid chromatography-tandem mass spectrometry (LC-MS), gas chromatography mass spectrometry (GC-MS), and the microbiological method have been applied for quantification of azoles. HPLC methods provide sufficient specificity and sensitivity for plasma VRZ concentration determination [6, 9].

Since, interindividual variability of the VRZ level and its related factors are still challenging, this study aimed to evaluate the relationship between VRZ plasma concentration and its efficacy and safety. A simple, rapid, specific, and sensitive HPLC-UV method for VRZ plasma concentration measurement was also developed.

## 2. Materials and Methods

### 2.1. Patients

Patients who initiated oral or IV VRZ for possible, probable, or proven invasive aspergillosis were included in the study. Definitions of proven, probable, and possible for the fungal disease were based on the revised European Organization for the Research and Treatment of Cancer/Mycosis Study Group (EORTC/MSG) 2008 consensus definition [13]. VRZ was administered as a loading dose of 6 mg/kg BD, followed by 4 mg/kg BD. The following data were recorded for all patients: gender, age, weight, BMI, route of administration, underlying diseases, outcome, adverse effects, and the biochemistry lab test. The evaluation of treatment as partial or complete success was based on clinical (fever, signs and symptoms of infection, and inflammatory markers) and radiological (CT or MRI findings) improvement or resolution and on proven or presumed eradication of the fungal pathogen. Failure to treatment was defined by persistent fungal infection after more than 14 days of treatment or by progressing fungal infection (clinical and radiological progression, persistently positive culture results, or death due to fungal infection) after more than seven days of treatment [14].

Exclusion criteria were the history of allergy or severe reaction to azoles, aspergilloma, or allergic bronchopulmonary aspergillosis, concomitant use of carbamazepine, efavirenz, rifampin, sirolimus, or ergot alkaloids that interact with VRZ, chronic invasive aspergillosis with duration of symptoms for more than four weeks, severe liver dysfunction (defined as total bilirubin, alanine transaminase (ALT), aspartate aminotransferase (AST), or alkaline phosphatase (ALP) > 3 times the upper limit of normal), and receiving antifungal therapy combination.

The study was approved by the ethics committee of Shahid Beheshti University of Medical Sciences with the ethics code of IR.SBMU.PHNM.1396.886.

### 2.2. Chemical and Instrumentation

VRZ powder source was a donation from Kish Medipharm Pharmaceutical Company. Clonazepam, used as internal standard, (IS) was obtained from Sobhan Pharmaceutical Company. HPLC grade acetonitrile, methanol, and ammonium acetate were acquired from Merck. The mobile phase composed of 0.05 M ammonium acetate/acetonitrile/methanol at 40 : 20 : 40 (v/v/v). The HPLC system consisted of Shimadzu pump model LC-10AD, coupled to a UV detector (model Shimadzu SPD-20A) adjusted at 256 nm. The separation was performed in the low-pressure gradient mode at 1 ml/min flow rate using a C_18_ column (250 mm × 4.6 mm with 3.5 *μ*m spherical particles).

### 2.3. Experimental

The stock solution of VRZ was prepared in methanol (100 *μ*g/ml). A series of seven calibrator solutions with concentrations of 2.5, 5, 10, 20, 40, 80, and 100 *μ*g/ml were prepared by VRZ stock solution in methanol. The stock solution of clonazepam was prepared in methanol (7.5 *μ*g/ml).

### 2.4. Sampling

For each patient, the blood sample was taken 30 minutes before the next dose under steady-state condition (minimum three days after VRZ administration) in the EDTA tubes. Blood samples were centrifuged at 3,000 rpm for 10 minutes to separate plasma. Plasma was kept in a microtube at −70°C until analysis time.

Blank plasma (450 *μ*l) was transferred to microcentrifuge tube; calibrator solution (50 *μ*l) and IS (250 *μ*l) were pipetted into it. Methanol (1250 *μ*l) was then added for protein precipitation. The suspension was vortexed for 2 minutes and then centrifuged at 10,000 rpm for 15 minutes. The supernatant was filtered with a syringe filter and then injected into the HPLC system.

The ratio of area under the curve (AUC) of the VRZ peak versus clonazepam peak was measured. These ratios were used to construct a calibration curve and to determine the VRZ concentration of patient samples using the calibration curve.

Three quality control (QC) samples with concentrations of 10, 50, and 80 *μ*g/ml were prepared in methanol and then added to the blank plasma to make final concentrations of 1, 5, and 8 *μ*g/ml. After addition of IS and methanol in the same proportion as calibrators, the samples were processed in the same manner as a calibrator.

The method for linearity, accuracy, and precision was validated. Accuracy and precision of interday and intraday were calculated by analyzing three QC samples with concentrations of 1, 5, and 8 *μ*g/ml. For intraday determination, these samples were measured in triplicates on the same day, and for interday determination, these samples were processed during three nonconsecutive days.

In order to analyze the patients' samples, each sample was first thawed at room temperature, and then, 500 *μ*l of it was transferred to a microcentrifuge tube. Finally, IS (250 *μ*l) and methanol (1250 *μ*l) were added to it. Tubes were capped and processed in the same manner as the calibrator.

### 2.5. Statistical Analysis

Analysis was performed using SPSS version 17.0 software (SPSS Inc., Chicago IL, US). We used appropriate descriptive statistics including median or mean and standard deviation (SD) for continuous variables and frequency and percentage for categorical variables. The Kolmogorov–Smirnov test was carried out to assess the normality of data distribution. Categorical variables were compared by the chi-square test. Parametric and nonparametric variables were compared by the ANOVA and Kruskal–Wallis test, respectively. *P* values <0.05 were considered significant.

## 3. Results

### 3.1. Patients

Twenty-four patients who started VRZ for invasive aspergillosis (14 proven, 9 probable, and 1 possible) were included (13 men and 11 women) in the study. Two of them were excluded because of concomitant use of efavirenz. Demographic characteristics and clinical data of the remaining twenty-two patients are presented in Table 1.

### 3.2. Peak Identification

IS was used to correct any possible error due to the protein precipitation and extraction of plasma samples. In this method, clonazepam was suitable as IS among several other compounds because of having similar logP to VRZ and no interaction with VRZ and plasma peaks.

Use of organic solvent (methanol) for the plasma protein precipitation is a rapid and simple way. We evaluated different proportions of methanol for this purpose. The most proper proportion was 3 : 1 methanol: plasma.

**Figure 1:**
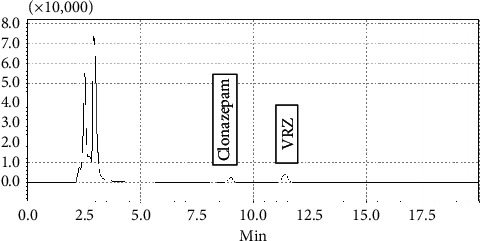
Chromatogram obtained from a patient on voriconazole (VRZ) therapy.

As shown in the chromatogram (Figure 1), by applying this protocol and chromatographic condition, retention time of clonazepam and VRZ was 9 and 11.5 minutes, respectively. Run time was 20 minutes, the resolution was good, and there was no overlapping of the peaks.

### 3.3. Linearity, LOD, and LOQ

**Figure 2:**
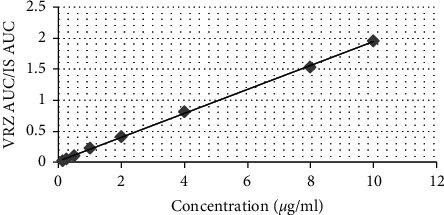
Linear relationship between area under the curve (AUC) of voriconazole/clonazepam peak ratio and plasma concentration of voriconazole over a range of 0.125–10 *μ*g/ml (VRZ, voriconazole; IS, internal standard).

There was a linear relationship between AUC of VRZ/clonazepam peak ratio and VRZ plasma concentration over a range of 0.125–10 *μ*g/ml, and the equation was *y*=0.192*x*+0.018 (*R*^2^ = 0.999). Calibration curve is shown in Figure 2.

LOQ was 0.125 *μ*g/ml, and LOD was estimated at a signal to noise ratio of 3 : 1, which was measured to be 0.042 *μ*g/ml.

### 3.4. Specification, Precision, and Accuracy

Blank plasma (without VRZ and clonazepam) did not present any interface with medication and IS peaks. Accuracy was ranged 85–115% for all concentrations. Interday and intraday precisions were within the acceptable limit of ±20% at the lower limit of quantification and ±15% of other concentrations.

### 3.5. VRZ Level in Plasma

The trough level was measured at a median of 5 days (range 3–8 days) after the initiation of treatment. Fifty percent of patients (11/22 patients) were out of the therapeutic range. From them, 27.27% and 22.73% were in subtherapeutic (<1 *μ*g/ml) and supratherapeutic (>5.5 *μ*g/ml) ranges, respectively.

### 3.6. VRZ Level and Outcome

Treatment failure in subtherapeutic and non-subtherapeutic levels was 83.33% and 18.75%, respectively.

### 3.7. VRZ Level and Toxicities

VRZ toxicities were observed more frequently in patients with supratherapeutic concentrations compared to non-supratherapeutic levels. Adverse reactions based on VRZ levels are presented in Table 2.

Furthermore, hepatic enzymes (ALT, AST, and ALP) and total bilirubin had more rises in the supratherapeutic group than the two other groups. Data are presented in Table 3.

### 3.8. VRZ Level and Mortality

Overall, 9 patients were expired. The rate of mortality was higher in patients with lower plasma concentration levels than those in therapeutic range (55.56% versus. 44.54%).

## 4. Discussion

In the present study, 50% of included patients had plasma levels which were out of the therapeutic range. Subtherapeutic and supratherapeutic levels were reported in 27.27% and 22.73% of patients, respectively. The results showed high variability in VRZ plasma concentration. Thus, the fixed-dose is not suitable for all patients. Other studies reported the same results in terms of plasma level variability. Chawla et al. suggested a large interindividual variability in VRZ levels ranging from subtherapeutic to toxic in 30% and 5% of patients, respectively [6]. Cabral-Galeano et al. reported 30.7% of cases who had out of therapeutic range levels (<1 *μ*g/ml in 19.2% and >5.5 *μ*g/ml in 11.5%) [11]. Based on Perreault et al. study, the percentage of the patients that achieved the therapeutic level increased significantly from 36% to 80% by applying the guideline of VRZ dose modification [15].

There was a significant statistic correlation between VRZ plasma concentration and clinical outcomes (*P*=0.022). Treatment failure in those with the subtherapeutic levels was 5 times higher than successful treatment, and also, failure to treatment in this group was 4.5 times higher than failure to treatment in patients with non-subtherapeutic levels. This result is in accordance with the meta-analysis that reported “patients with therapeutic voriconazole serum concentration were twice as likely to achieve a successful outcome” [8].

Although, mortality rate was not statistically associated with VRZ levels (*P*=0.609) in patients with failure to treatment, it was 2.17 times higher than its rate in patients with successful treatment (62.50% versus. 28.57%). Miyakis et al. showed that patients with the initial VRZ trough level ≤0.35 *μ*g/ml had significantly an 11-fold increase in the risk of death compared to patients who had the plasma concentration level above 0.35 *μ*g/ml [16].

Based on other studies, many factors can influence the VRZ level such as age, sex, weight, underlying diseases, drug interactions, and genetic polymorphism [8, 11, 12]. In this study, there was no significant relationship between VRZ plasma concentration and age (*P*=0.389), sex (*P*=0.676), BMI (*P*=0.363), and route of administration (*P*=0.142). We did not assess genetic polymorphism, as a limitation of our study. Obeng et al. reported that CYP2C19 genotype has an impact on trough concentration of VRZ [17].

Based on Vanstraelen et al. study, if plasma albumin decreases by 1 g/dl and bilirubin increases by 1 g/dl, VRZ protein binding decreases by 0.67% and 0.19%, respectively [18]. In our study, the patients with supratherapeutic levels had lower albumin levels compared to the therapeutic and subtherapeutic groups (1.41 versus 2.87 and 3.47 g/dl). They also had higher bilirubin levels than the therapeutic and the subtherapeutic groups (1.5 versus 0.95 and 0.40 g/dl). Hypoalbuminemia and hyperbilirubinemia may influence the VRZ protein binding as well as the plasma level [18].

Many methods have been used for VRZ plasma concentration measurement; each has its advantages and disadvantages. One of the methods is bioassay which is reliable for VRZ level measurement, but it has some limitations, including inability to identify the difference between drug and active metabolite, effect of combination antifungal therapy on inhibition growth zone, and time-consuming process of running the test. Badiee et al. reported that bioassay can be used for determining VRZ plasma concentration when the HPLC method is not applied in laboratories [9]. The other method that was used for this purpose is UPLC-MS/MS. It is a simple and rapid method for monitoring triazole antifungals but is not easily accessible [19]. Prommas et al. used LC-MS-MS with fast and accurate runtime, but it is costly and not available in all laboratories [20].

The HPLC-UV method that we used was rapid, simple, and had the advantage of convenient sampling. It can be used in medical centers easily and provides sufficient specificity and selectivity for VRZ plasma concentration measurement.

The limitations of our study were the small sample size, not assessing the genetic polymorphism, and the short follow-up period of the patients.

## 5. Conclusion

VRZ plasma level had a considerable interindividual variability. TDM of VRZ can play an important role in providing a higher efficacy treatment of invasive aspergillosis with lower toxicity. Determination of the drug level may have clinical benefits.

## Figures and Tables

**Table 1:** Patients' demographic characteristics and clinical data.

Characteristics	Number (%)
Male sex	12 (54.54)
Age	Median: 57 years (range: 21–69 years)
Weight	Median: 61 kg (range: 36–90 kg)
BMI	Median: 21.61 kg/m^2^ (range: 14.98–37.45 kg/m^2^)
Infection	
Proven	12 (54.54)
Probable	9 (40.91)
Possible	1 (4.55)
Underlying diseases	
DM	2 (9.09)
HTN	5 (22.73)
COPD	4 (18.18)
CVA	1 (4.55)
CKD	3 (13.64)
Chronic steroid use	6 (27.27)
Hypothyroidism	3 (13.64)
Route of administration	
Oral	17 (77.27)
IV	3 (13.64)
IV to oral	2 (9.09)

DM, diabetes mellitus; HTN, hypertension; COPD, chronic obstructive pulmonary disease; CVA, cerebrovascular accident; CKD, chronic kidney disease.

**Table 2:** Frequency of adverse reactions according to voriconazole plasma levels.

Adverse reactions	VRZ levels			Total	*P* value
	Subtherapeutic	Therapeutic	Supratherapeutic		
Skin rash	0	1	2	3	0.129
Tachycardia	0	0	1	1	0.168
Fever	0	2	3	5	0.054
Nausea and vomiting	1	0	2	3	0.094

**Table 3:** The results of the liver function test according to voriconazole plasma levels.

	VRZ levels			
	Subtherapeutic	Therapeutic	Supratherapeutic	*P* value
Mean of total bilirubin (mg/dl)	0.4 ± 0.26	0.95 ± 0.70	1.5 ± 1.13	0.161
Mean of ALT (U/L)	35.33 ± 27.02	84.5 ± 79.08	981 ± 136.13	0.163
Mean of AST (U/L)	22.33 ± 8.39	96.67 ± 88.74	915 ± 123.85	0.151
Mean of ALP (U/L)	228.67 ± 21.82	555 ± 50.05	764.17 ± 87.03	0.210

ALT, alanine transaminase; AST, aspartate aminotransferase; ALP, alkaline phosphatase.

## Data Availability

The data used to support the findings of this study are available from the corresponding author upon request.
